# Epidemics after Natural Disasters

**DOI:** 10.3201/eid1301.060779

**Published:** 2007-01

**Authors:** John T. Watson, Michelle Gayer, Maire A. Connolly

**Affiliations:** * World Health Organization, Geneva, Switzerland

**Keywords:** Disasters, epidemiology, outbreaks, surveillance, risk assessment, communicable diseases, perspective

## Abstract

The relationship between natural disasters and communicable diseases is frequently misconstrued. The risk for outbreaks is often presumed to be very high in the chaos that follows natural disasters, a fear likely derived from a perceived association between dead bodies and epidemics. However, the risk factors for outbreaks after disasters are associated primarily with population displacement. The availability of safe water and sanitation facilities, the degree of crowding, the underlying health status of the population, and the availability of healthcare services all interact within the context of the local disease ecology to influence the risk for communicable diseases and death in the affected population. We outline the risk factors for outbreaks after a disaster, review the communicable diseases likely to be important, and establish priorities to address communicable diseases in disaster settings.

Natural disasters are catastrophic events with atmospheric, geologic, and hydrologic origins. Disasters include earthquakes, volcanic eruptions, landslides, tsunamis, floods, and drought. Natural disasters can have rapid or slow onset, with serious health, social, and economic consequences. During the past 2 decades, natural disasters have killed millions of people, adversely affected the lives of at least 1 billion more people, and resulted in substantial economic damages (*1*). Developing countries are disproportionately affected because they lack resources, infrastructure, and disaster-preparedness systems.

Deaths associated with natural disasters, particularly rapid-onset disasters, are overwhelmingly due to blunt trauma, crush-related injuries, or drowning. Deaths from communicable diseases after natural disasters are less common.

## Dead Bodies and Disease

The sudden presence of large numbers of dead bodies in the disaster-affected area may heighten concerns of disease outbreaks (*2*), despite the absence of evidence that dead bodies pose a risk for epidemics after natural disasters (*3*). When death is directly due to the natural disaster, human remains do not pose a risk for outbreaks (*4*). Dead bodies only pose health risks in a few situations that require specific precautions, such as deaths from cholera (*5*) or hemorrhagic fevers (*6*). Recommendations for management of dead bodies are summarized in the Table.

**Table:** Principles for management of dead bodies*

• Mass management of dead bodies is often based on the false belief that they represent an epidemic hazard if not buried or burned immediately.
• Burial is preferable to cremation in mass casualty situations.
• Every effort should be made to identify the bodies. Mass burial should be avoided if at all possible.
• Families should have the opportunity (and access to materials) to conduct culturally appropriate funerals and burials according to social custom.
• Where existing facilities such as graveyards or crematoria are inadequate, alternative locations or facilities should be provided.
• For workers routinely handling bodies, ensure
• Universal precautions for blood and body fluids
• Use and correct disposal of gloves
• Use of body bags if available
• Hand-washing with soap after handling bodies and before eating
• Disinfection of vehicles and equipment
• Bodies do not need disinfection before disposal (except in cases of cholera, shigellosis, or hemorrhagic fever)
• Bottom of any grave is >1.5 m above the water table, with a 0.7-m unsaturated zone

*Adapted from Morgan (*3*).

Despite these facts, the risk for outbreaks after disasters is frequently exaggerated by both health officials and the media. Imminent threats of epidemics remain a recurring theme of media reports from areas recently affected by disasters, regardless of attempts to dispel these myths (*2*,*3*,*7*).

## Displacement: Primary Concern

The risk for communicable disease transmission after disasters is associated primarily with the size and characteristics of the population displaced, specifically the proximity of safe water and functioning latrines, the nutritional status of the displaced population, the level of immunity to vaccine-preventable diseases such as measles, and the access to healthcare services (*8*). Outbreaks are less frequently reported in disaster-affected populations than in conflict-affected populations, where two thirds of deaths may be from communicable diseases (*9*). Malnutrition increases the risk for death from communicable diseases and is more common in conflict-affected populations, particularly if their displacement is related to long-term conflict (*10*).

Although outbreaks after flooding (*11*) have been better documented than those after earthquakes, volcanic eruptions, or tsunamis (*12*), natural disasters (regardless of type) that do not result in population displacement are rarely associated with outbreaks (*8*). Historically, the large-scale displacement of populations as a result of natural disasters is not common (*8*), which likely contributes to the low risk for outbreaks overall and to the variability in risk among disasters of different types.

## Risk Factors for Communicable Disease Transmission

Responding effectively to the needs of the disaster-affected population requires an accurate communicable disease risk assessment. The efficient use of humanitarian funds depends on implementing priority interventions on the basis of this risk assessment.

A systematic and comprehensive evaluation should identify 1) endemic and epidemic diseases that are common in the affected area; 2) living conditions of the affected population, including number, size, location, and density of settlements; 3) availability of safe water and adequate sanitation facilities; 4) underlying nutritional status and immunization coverage among the population; and 5) degree of access to healthcare and to effective case management.

## Communicable Diseases Associated with Natural Disasters

The following types of communicable diseases have been associated with populations displaced by natural disasters. These diseases should be considered when postdisaster risk assessments are performed.

### Water-related Communicable Diseases

Access to safe water can be jeopardized by a natural disaster. Diarrheal disease outbreaks can occur after drinking water has been contaminated and have been reported after flooding and related displacement. An outbreak of diarrheal disease after flooding in Bangladesh in 2004 involved >17,000 cases; *Vibrio cholerae* (O1 Ogawa and O1 Inaba) and enterotoxigenic *Escherichia coli* were isolated (*13*). A large (>16,000 cases) cholera epidemic (O1 Ogawa) in West Bengal in 1998 was attributed to preceding floods (*14*), and floods in Mozambique in January–March 2000 led to an increase in the incidence of diarrhea (*15*).

In a large study undertaken in Indonesia in 1992–1993, flooding was identified as a significant risk factor for diarrheal illnesses caused by *Salmonella enterica* serotype Paratyphi A (paratyphoid fever) (*16*). In a separate evaluation of risk factors for infection with *Cryptosporidium parvum* in Indonesia in 2001–2003, case-patients were >4× more likely than controls to have been exposed to flooding (*17*).

The risk for diarrheal disease outbreaks following natural disasters is higher in developing countries than in industrialized countries (*8*,*11*). In Aceh Province, Indonesia, a rapid health assessment in the town of Calang 2 weeks after the December 2004 tsunami found that 100% of the survivors drank from unprotected wells and that 85% of residents reported diarrhea in the previous 2 weeks (*18*). In Muzaffarabad, Pakistan, an outbreak of acute watery diarrhea occurred in an unplanned, poorly equipped camp of 1,800 persons after the 2005 earthquake. The outbreak involved >750 cases, mostly in adults, and was controlled after adequate water and sanitation facilities were provided (*19*). In the United States, diarrheal illness was noted after Hurricanes Allison (*20*) and Katrina (*21*–*23*), and norovirus, *Salmonella*, and toxigenic and nontoxigenic *V. cholerae* were confirmed among Katrina evacuees.

Hepatitis A and E are also transmitted by the fecal-oral route, in association with lack of access to safe water and sanitation. Hepatitis A is endemic in most developing countries, and most children are exposed and develop immunity at an early age. As a result, the risk for large outbreaks is usually low in these settings. In hepatitis E–endemic areas, outbreaks frequently follow heavy rains and floods; the illness is generally mild and self-limited, but in pregnant women case-fatality rates can reach 25% (*24*). After the 2005 earthquake in Pakistan, sporadic hepatitis E cases and clusters were common in areas with poor access to safe water. Over 1,200 cases of acute jaundice, many confirmed as hepatitis E, occurred among the displaced (*25*). Clusters of both hepatitis A and hepatitis E were noted in Aceh after the December 2004 tsunami (*26*).

Leptospirosis is an epidemic-prone zoonotic bacterial disease that can be transmitted by direct contact with contaminated water. Rodents shed large amounts of leptospires in their urine, and transmission occurs through contact of the skin and mucous membranes with water, damp soil or vegetation (such as sugar cane), or mud contaminated with rodent urine. Flooding facilitates spread of the organism because of the proliferation of rodents and the proximity of rodents to humans on shared high ground. Outbreaks of leptospirosis occurred in Taiwan, Republic of China, associated with Typhoon Nali in 2001 (*27*); in Mumbai, India, after flooding in 2000 (*28*); in Argentina after flooding in 1998 (*29*); and in the Krasnodar region of the Russian Federation in 1997 (*30*). After a flooding-related outbreak of leptospirosis in Brazil in 1996, spatial analysis indicated that incidence rates of leptospirosis doubled inside the flood-prone areas of Rio de Janeiro (*31*).

### Diseases Associated with Crowding

Crowding is common in populations displaced by natural disasters and can facilitate the transmission of communicable diseases. Measles and the risk for transmission after a natural disaster are dependent on baseline immunization coverage among the affected population, and in particular among children <15 years of age. Crowded living conditions facilitate measles transmission and necessitate even higher immunization coverage levels to prevent outbreaks (*32*). A measles outbreak in the Philippines in 1991 among persons displaced by the eruption of Mt. Pinatubo involved >18,000 cases (*33*). After the tsunami in Aceh, a cluster of measles involving 35 cases occurred in Aceh Utara district, and continuing sporadic cases and clusters were common despite mass vaccination campaigns (*26*). In Pakistan, after the 2005 South Asia earthquake, sporadic cases and clusters of measles (>400 clinical cases in the 6 months after the earthquake) also occurred (*25*).

*Neisseria meningitidis* meningitis is transmitted from person to person, particularly in situations of crowding. Cases and deaths from meningitis among those displaced in Aceh and Pakistan have been documented (*25*,*26*). Prompt response with antimicrobial prophylaxis, as occurred in Aceh and Pakistan, can interrupt transmission. Large outbreaks have not been recently reported in disaster-affected populations but are well-documented in populations displaced by conflict (*34*).

Acute respiratory infections (ARI) are a major cause of illness and death among displaced populations, particularly in children <5 years of age. Lack of access to health services and to antimicrobial agents for treatment further increases the risk for death from ARI. Risk factors among displaced persons include crowding, exposure to indoor cooking using open flame, and poor nutrition. The reported incidence of ARI increased 4-fold in Nicaragua in the 30 days after Hurricane Mitch in 1998 (*35*), and ARI accounted for the highest number of cases and deaths among those displaced by the tsunami in Aceh in 2004 (*26*) and by the 2005 earthquake in Pakistan (*25*).

### Vectorborne Diseases

Natural disasters, particularly meteorologic events such as cyclones, hurricanes, and flooding, can affect vector-breeding sites and vectorborne disease transmission. While initial flooding may wash away existing mosquito-breeding sites, standing water caused by heavy rainfall or overflow of rivers can create new breeding sites. This situation can result (with typically some weeks’ delay) in an increase of the vector population and potential for disease transmission, depending on the local mosquito vector species and its preferred habitat. The crowding of infected and susceptible hosts, a weakened public health infrastructure, and interruptions of ongoing control programs are all risk factors for vectorborne disease transmission (*36*).

Malaria outbreaks in the wake of flooding are a well-known phenomenon. An earthquake in Costa Rica’s Atlantic Region in 1991 was associated with changes in habitat that were beneficial for breeding and preceded an extreme rise in malaria cases (*37*). Additionally, periodic flooding linked to El Niño–Southern Oscillation has been associated with malaria epidemics in the dry coastal region of northern Peru (*38*).

Dengue transmission is influenced by meteorologic conditions, including rainfall and humidity, and often exhibits strong seasonality. However, transmission is not directly associated with flooding. Such events may coincide with periods of high risk for transmission and may be exacerbated by increased availability of the vector’s breeding sites (mostly artificial containers) caused by disruption of basic water supply and solid waste disposal services. The risk for outbreaks can be influenced by other complicating factors, such as changes in human behavior (increased exposure to mosquitoes while sleeping outside, movement from dengue-nonendemic to -endemic areas, a pause in disease control activities, overcrowding) or changes in the habitat that promote mosquito breeding (landslide, deforestation, river damming, and rerouting of water).

### Other Diseases Associated with Natural Disasters

Tetanus is not transmitted person to person but is caused by a toxin released by the anaerobic tetanus bacillus *Clostridium tetani*. Contaminated wounds, particularly in populations where vaccination coverage levels are low, are associated with illness and death from tetanus. A cluster of 106 cases of tetanus, including 20 deaths, occurred in Aceh and peaked 2-1/2 weeks after the tsunami (*26*). Cases were also reported in Pakistan following the 2005 earthquake (*25*).

An unusual outbreak of coccidiomycosis occurred after the January 1994 Southern California earthquake. The infection is not transmitted person to person and is caused by the fungus *Coccidioides immitis*, which is found in soil in certain semiarid areas of North and South America. This outbreak was associated with exposure to increased levels of airborne dust subsequent to landslides in the aftermath of the earthquake (*39*).

### Disaster-Related Interruption of Services

Power cuts related to disasters may disrupt water treatment and supply plants, thereby increasing the risk for waterborne diseases. Lack of power may also affect proper functioning of health facilities, including preservation of the vaccine cold chain. An increase in diarrheal illness in New York City followed a massive power outage in 2003. The blackout left 9 million people in the area without power for several hours to 2 days. Diarrhea cases were widely dispersed and detected by using nontraditional surveillance techniques. A case-control study performed as part of the outbreak investigation linked diarrheal illness with the consumption of meat and seafood after the onset of the power outage, when refrigeration facilities were widely interrupted (*40*).

## Discussion

Historically, fears of major disease outbreaks in the aftermath of natural disasters have shaped the perceptions of the public and policymakers. These expectations, misinformed by associations of disease with dead bodies, can create fear and panic in the affected population and lead to confusion in the media and elsewhere.

The risk for outbreaks after natural disasters is low, particularly when the disaster does not result in substantial population displacement. Communicable diseases are common in displaced populations that have poor access to basic needs such as safe water and sanitation, adequate shelter, and primary healthcare services. These conditions, many favorable for disease transmission, must be addressed immediately with the rapid reinstatement of basic services. Assuring access to safe water and primary healthcare services is crucial, as are surveillance and early warning to detect epidemic-prone diseases known to occur in the disaster-affected area. A comprehensive communicable disease risk assessment can determine priority diseases for inclusion in the surveillance system and prioritize the need for immunization and vector-control campaigns. Five basic steps that can reduce the risk for communicable disease transmission in populations affected by natural disasters are summarized in an (Appendix Table).

Disaster-related deaths are overwhelmingly caused by the initial traumatic impact of the event. Disaster-preparedness plans, appropriately focused on trauma and mass casualty management, should also take into account the health needs of the surviving disaster-affected populations. The health effects associated with the sudden crowding of large numbers of survivors, often with inadequate access to safe water and sanitation facilities, will require planning for both therapeutic and preventive interventions, such as the rapid delivery of safe water and the provision of rehydration materials, antimicrobial agents, and measles vaccination materials.

Surveillance in areas affected by disasters is fundamental to understanding the impact of natural disasters on communicable disease illness and death. Obtaining relevant surveillance information in these contexts, however, is frequently challenging. The destruction of the preexisting public health infrastructure can aggravate (or eliminate) what may have been weak predisaster systems of surveillance and response. Surveillance officers and public health workers may be killed or missing, as in Aceh in 2004. Population displacement can distort census information, which makes the calculation of rates for comparison difficult. Healthcare during the emergency phase is often delivered by a wide range of national and international actors, which creates coordination challenges. Also, a lack of predisaster baseline surveillance information can lead to difficulties in accurately differentiating epidemic from background endemic disease transmission.

Although postdisaster surveillance systems are designed to rapidly detect cases of epidemic-prone diseases, interpreting this information can be hampered by the absence of baseline surveillance data and accurate denominator values. Detecting cases of diseases that occur endemically may be interpreted (because of absence of background data) as an early epidemic. The priority in these settings, however, is rapid implementation of control measures when cases of epidemic-prone diseases are detected. Despite these challenges, continued detection of and response to communicable diseases are essential to monitor the incidence of diseases, to document their effect, to respond with control measures when needed, and to better quantify the risk for outbreaks after disasters.

## Supplementary Material

Appendix Table Priority measures to reduce the risk for communicable diseases after natural disasters
