# Supplementary material for: Epidemics after Natural Disasters
Source: Emerg Infect Dis. 2007 Jan;13(1):1–5. doi: 10.3201/eid1301.060779 (PMC2725828; doi:10.3201/eid1301.060779)
Supplement: Appendix Table — Priority measures to reduce the risk for communicable diseases after natural disasters [file 06-0779_appT-s1.pdf]

**Appendix Table.** Priority measures to reduce the risk for communicable diseases after natural disasters

---

1. Safe water, sanitation, site planning

Ensuring uninterrupted provision of safe drinking water is the most important preventive measure to be implemented following a natural disaster. Chlorine is widely available, inexpensive, easily used, and effective against nearly all waterborne pathogens.

Settlement planning must provide for adequate access for water and sanitation needs and meet the minimum space requirements per person, in accordance with international guidelines.

---

2. Primary healthcare services

Access to primary care is critical for prevention, early diagnosis, and treatment of a wide range of diseases, as well as for providing an entry point for secondary and tertiary care. The immediate impact of communicable diseases can be mitigated with the following interventions:

- Early diagnosis and treatment of diarrheal diseases and ARI,* particularly in those aged <5 y.
- Early diagnosis and treatment for malaria in malaria-endemic areas (within 24 h of onset of fever, using artemisinin-based combination therapy for falciparum malaria).
- Availability and use of treatment protocols for the main communicable disease threats.
- Proper wound cleaning and care. Tetanus toxoid with or without tetanus immunoglobulin, as appropriate, should accompany wound treatment postdisaster.
- Availability of drugs included in the interagency emergency health kit,† e.g., oral rehydration salts for management of diarrheal diseases, antimicrobial agents for ARI.
- Distribution of health education messages emphasizing:

    Good hand hygiene practices

    Safe food preparation techniques

    Boiling or chlorination of water

    Early treatment seeking behavior in case of fever

    Use of insecticide-treated mosquito nets as a personal protection measure in malaria-endemic areas

    Vector control interventions adapted to the local context and disease epidemiology

---

3. Surveillance/early warning system

Rapid detection of cases of epidemic-prone diseases is essential to ensure rapid control. A surveillance/early warning system should be quickly established to detect outbreaks and monitor priority endemic diseases.

- Priority diseases to be included in surveillance system should be based on a systematic communicable disease risk assessment.
- Healthcare workers should be trained to detect priority diseases and promptly report them to lead health agency.
- Sampling and transport materials (and appropriate stockpiles) for investigation need to be readily available for rapid response to outbreaks, e.g., cholera kits, if cholera is considered a risk.

---

4. Immunization

Mass measles immunization and vitamin A supplementation are immediate health priorities in areas with inadequate coverage. Where baseline coverage rates among those <15 y of age are<90%, mass measles immunization should be implemented as soon as possible. The priority age groups are 6 mo to 5 y, and up to 15 y, if resources allow.

Current typhoid vaccines are not recommended for mass campaigns to prevent typhoid disease. Typhoid vaccination in conjunction with other preventive measures may be useful to control typhoid outbreaks, depending on local circumstances.

Hepatitis A vaccine is generally not recommended to prevent outbreaks in the disaster area.

The cost of the cholera vaccine and the logistic difficulties involved with its administration have prohibited its widespread use. Although helpful in specific circumstances, it should not be viewed as a replacement for adequate water and sanitation. The usefulness of the cholera vaccine, relative to other public health priorities, has not been evaluated in disaster-affected areas.

---

5. Prevention of malaria and dengue

Specific preventive interventions for malaria must be based on an informed assessment of the local situation, including on the prevalent parasite species and the main vectors.

An increase in mosquito numbers may be delayed following flooding, which allows time for implementing preventive measures such as indoor residual spraying of insecticides, or the retreatment/distribution of insecticide-treated nets, preferably long-lasting insecticidal nets in areas where their use is well-known and accepted.

Weekly case numbers must be monitored to allow early detection of malaria outbreaks. Periodic laboratory confirmation of rapid test–positive fever cases is recommended to track the slide/test positivity rate.

Treatment with artemisinin-based combination therapy should be provided free of charge to the user in areas with falciparum malaria. An active search for fever cases may be necessary to reduce deaths.

For dengue, the main preventive efforts should be directed toward vector control. Social mobilization and health education of the community should emphasize elimination of breeding sites as much as possible, specifically by:

- Continuous covering of all stored water containers
- Removal or destruction of solid debris where water can collect (e.g., bottles, tires, tins).

---

*ARI, acute respiratory infections.

†Available from https://www.who.int/medicines/publications/mrhealthkit.pdf
